# Improving energy sustainability for public buildings in Italian mountain communities

**DOI:** 10.1016/j.heliyon.2018.e00628

**Published:** 2018-05-14

**Authors:** Guglielmina Mutani, Mauro Cornaglia, Massimo Berto

**Affiliations:** a Department of Energy, Politecnico di Torino, Torino (IT), Italy; b Environment Park, Torino (IT), Italy; c Sertec Engineering Consulting, Torino (IT), Italy

**Keywords:** Energy, Environmental science

## Abstract

The objective of this work is to analyze and then optimize thermal energy consumptions of public buildings located within the mountain community of Lanzo, Ceronda and Casternone Valleys. Some measures have been proposed to reduce energy consumption and consequently the economic cost for energy production, as well as the harmful GHG emissions in the atmosphere.

Initially, a study of the mountain territory has been carried out, because of its vast extension and climatic differences. Defined the communities and the buildings under investigation, energy dependant data were collected for the analysis of energy consumption monitoring: consumption data of three heating seasons, geometric buildings characteristics, type of opaque and transparent envelope, heating systems information with boiler performance and climatic data.

Afterward, five buildings with critical energy performances were selected; for each of these buildings, different retrofit interventions have been hypothesized to reduce the energy consumption, with thermal insulation of vertical or horizontal structures, new windows or boiler substitution. The cost-optimal technique was used to choose the interventions that offered higher energy performance at lower costs; then a retrofit scenario has been planned with a specific financial investment.

Finally, results showed possible future developments and scenarios related to buildings energy efficiency with regard to the topic of biomass exploitation and its local availability in this area. In this context, the biomass energy resource could to create a virtuous environmental, economic and social process, favouring also local development.

## 1 Introduction

During the last half of the century, worldwide matters as climatic change, global warming and limited resource depletion became more current and urgent. These issues are strictly related to the emission of greenhouse gases (GHG) coming from anthropic activities. In order to reduce the negative effect of climate change, global warming and limited resource depletion over economic, social and natural systems, a rapid decrease in GHG emissions is needed, together with policies and strategies for a more sustainable and resilient society. In particular, energy resilience implies a functioning and stable energy system, providing continuity and minimizing service interruptions; then, energy security and sustainability are among the most important aspects in urban energy resilience [1].

The energy sector is still responsible for about 60% of global GHG emissions, and therefore it is indisputable that the sustainability of the environment is indissolubly influenced by the energy sector [2]. Therefore, energy sector should be focused on the opportunities and challenges to ensure accessible, reliable, affordable and clean energy sources, also stimulating the economic growth, social welfare and job creation.

In Italy, to improve energy security and sustainability, prior actions are energy efficiency and the use of renewable energy technologies, reducing also energy imports from abroad; these actions are encouraged also by public subsides. The climate and energy targets of EU are indeed attainable through an energy transition of human activities from non-renewable to renewable energy sources associated with more energy efficiency.

The civil sector plays a central role accounting for 37.1% of final energy consumption in the EU with a steady growth of +33.8% over the 1994–2014 period [3]. It is therefore urgent to intervene on private and public buildings energy consumptions in order to improve their energy performance, reducing their needs. Also, the EU 2030 Strategy encourages its members to take actions on energy efficiency especially in the existing building heritage and in Italy, policies of financial incentives have been provided to encourage investments in civil sector with retrofit interventions and producing energy with renewable sources.

The aim of this work is to define a methodology through a particular case study: improving energy sustainability of public buildings in a mountain community. After the description of the case study, in the norther part of Italy, energy consumptions and the available renewable energy sources have been mapped with the use of a Geographic Information System (GIS). In particular, the proposed GIS-based approach provides a localized optimization of energy demand and supply, exploiting the available wooden biomass resources near the area for a sustainable, secure and replicable energy planning.

## 2 Background

For this research work, the scientific literature has been investigated, in particular articles related to energy consumption monitoring and energy retrofit of buildings. Semprini et al. [4] used the energy signature to analyse energy consumptions, proposing three not invasive and low cost scenarios. Ma et al. [5] proposed a methodology for a wide building heritage, dividing the buildings by type of user, thermal vector, system efficiencies, period of construction, geometry and type of envelope. Yaquin et al. [6] focused their work on the reduction of energy consumption with energy saving measures, highlighting the importance of the real energy needs of users. Mutani et al. [7, 8] implemented energy consumption data and building characteristics with a GIS tool for 50 municipalities near Turin, comparing the results of bottom-up and top-down models from building scale to territorial scale. Rospi et al. [9] compared the measured and estimated energy consumptions of a building with energy requirements. Finally, Aelenei et al. [10] and Mutani et al. [11] evaluated the nZEB for European countries considering the application of the European Directives, for a reduction of energy consumption in the building sector considering any political or economic obstacles that could reduce the strategic impact.

In this work, two GIS-based methodologies are associated: the first to identify the critical buildings that consume more energy for space heating, where priority of intervention should be applied [12]; the second one to evaluate, with a cost-benefit analysis, a priority order of the interventions to be implemented from an economic and environmental point of view [13]. In addition, these methodologies have been adapted to a very various building heritage on a very vast territorial area with different climatic characteristics; finally, the cost-optimal analysis was also modified to favour renewable energy sources. These methodologies have the advantage to be replicable in unions of municipalities, as the analysed mountain community, and in energy communities for which an approval process of a law is under way in the Piedmont Region.

## 3 Study area

### 3.1 Study area of Lanzo, Ceronda and Casternone Valleys Union

The geographical area of this case study is the mountainous territory at North-West of Turin and in the North-West part of Italy and it is composed by 21 municipalities administered by a public entity named Mountain Union of Lanzo, Ceronda and Casternone Valleys (in Italian: Unione Montana Valli di Lanzo, Ceronda e Casternone U.M.V.L.C.C.). This territory is crossed by Stura di Lanzo river and it can be subdivided in 4 valleys: two main ones (Val Grande and Val d'Ala) and two minor ones (Ceronda and Casternone pre-Alpine valleys).

This mountain community is located in an area of approximately 478 km^2^ at 50 km from the city of Turin. Each municipality has an average extension of 22 km^2^ with maximum values of 62 and 46 km^2^, respectively for Balme and Ala di Stura, and minimum values of 5 and 6 km^2^ for Pessinetto and Vallo Torinese. From a demographic point of view, the average population is of 1742 inhabitants per municipality: the most populated is Lanzo Torinese with 5133 inhabitants, while the less populated is Balme with only 112 inhabitants. In this context, public buildings are sprawled on the territory with very different characteristics both by typology and size and then, it is not possible to find recurring buildings archetypes in terms of geometric shape, structural characteristics and materials used.

**Fig. 1:**
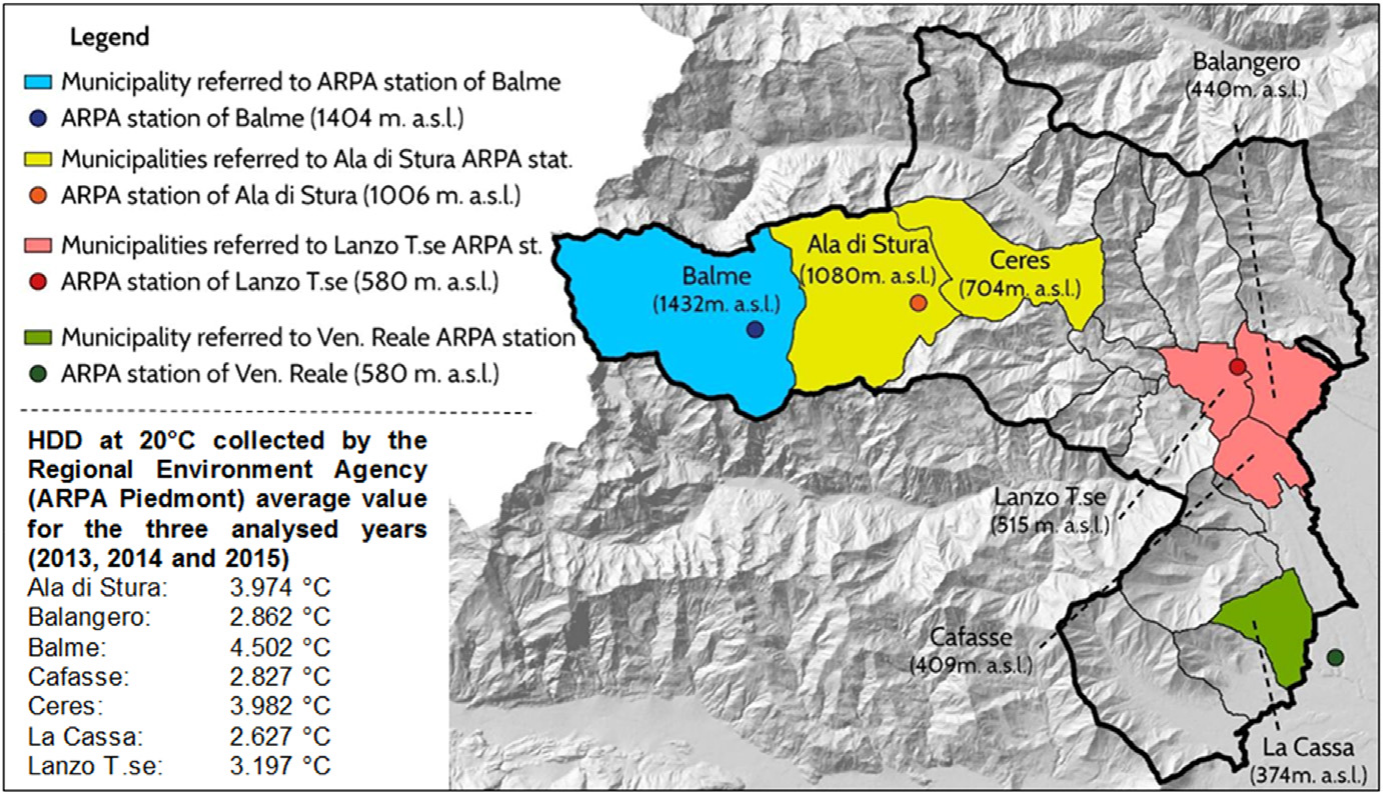
Municipalities investigated by the survey with its meteorological stations.

In order to obtain a representative sample, seven municipalities have been chosen with various climatic and topographic characteristics, different number of inhabitants and dimensions, as shown in Fig. 1: three little municipalities in the high valley (Balme, Ala di Stura and Ceres), three bigger municipalities in the medium valley (Lanzo Torinese, Balangero and Cafasse) and one in low valley (La Cassa).

Topographic differences, especially altitude and valley orientation, influence climate characteristics and then different databases on daily air temperatures and Heating Degree Days at 20 °C (HDD) have been collected by the Regional Environment Agency (ARPA Piemonte) weather stations (in Fig. 1). For each municipality a weather station has been identified, geographically close to the municipality and with similar altitude; climate data have been collected for three years: 2013, 2014 and 2015. The area considered in this study is characterized by a climatic heterogeneity, presenting average temperatures that vary of about 4.5 °C in coldest month and 7.6 °C in the hottest month between Balme (at 1410 m a.s.l.) and Venaria Reale (at 337 m a.s.l.), as shown in Table 1. This heterogeneity of climate is also evident in the HDD values recorded by the different weather stations, with a 2013 colder than the other years and with high differences between the weather stations as in 2014 with 4768 HDD registered by Balme and 2453 HDD registered by Venaria Reale in the same year.

**Table 1:** Average monthly temperatures and HDD at 20 °C registered by the weather stations for the years 2013, 2014, 2015.

	Year	Jan	Feb	Mar	Apr	May	June	July	Aug	Sep	Oct	Nov	Dec	HDD
**Ala di Stura**^a^ (1006 m a.s.l.)	**2013**	−0.9	−1.8	2.2	7.6	9.6	14.6	17.8	16.8	13.5	8.8	2.9	−0.4	4523
	**2014**	−1	0.5	5.2	9.3	10.9	15.2	15.6	15.1	13.6	10	4.8	0.9	4237
	**2015**	−0.1	−0.5	4.2	9	12.6	15.8	20	16.8	11.8	7.5	4	0.7	4204
**Lanzo Torinese**^b^ (580 m a.s.l.)	**2013**	2.6	1.4	5.2	10.7	12.9	18.7	21.9	20.5	16.7	11.8	6.5	3.8	3366
	**2014**	3	4.1	8.8	12.4	14.4	19.1	19.3	18.8	16.7	13.3	8.2	4.3	3006
	**2015**	3.7	2.8	7.8	12.2	16.1	20	24.6	20.8	15.5	10.8	8.1	5.2	3019
**Balme**^c^ (1410 m a.s.l.)	**2013**	−0.9	−3.2	0.4	5.2	7.4	12.5	15.9	14.8	11.9	7.1	1.7	−0.1	5072
	**2014**	−1.5	−0.7	3.7	6.9	8.9	13.1	13.8	13.2	11.7	6.3	3.5	0.9	4768
	**2015**	0.1	−1.6	2.9	6.9	10.7	13.9	18.1	14.9	9.7	5.9	5.7	1.3	4593
**Venaria Reale**^d^ (337 m a.s.l.)	**2013**	1.8	0.9	5.8	11.8	14.1	20	23.3	21.8	17.9	12.8	6.8	2.1	2832
	**2014**	2.9	4.9	9	13.1	15.4	20.6	20.7	20.3	17.8	14.1	8.5	3.6	2453
	**2015**	2.4	3	8.5	12.6	17.4	21.5	25.9	22.2	16.8	11.7	6.8	3.4	2482

a Nearest weather station for the municipalities of Ceres and Ala di Stura.

b Nearest weather station for the municipalities of Lanzo Torinese, Cafasse e Balangero.

c Nearest weather station for the municipality of Balme.

d Nearest weather station for the municipality of La Cassa.

Within the selected municipalities, 48 buildings were chosen to represent the public building heritage. The chosen buildings were characterized by different period of construction, type of user and energy system. About 60% of the buildings were built before 1976 (before the first Italian Law on building energy performance L.373/1976; 27% are schools, 24% are used for entertainment activities, and 23% are offices). About the heating systems, 67% are heated with natural gas, while 20% still uses gas oil. The selection of the public buildings for this analysis included also lacks in information on the type of use and data about energy consumptions; in the mountains, some buildings can be used with discontinuity and with various types of heating systems. This analysis excluded 12 public buildings for lack of data and then the analysed buildings were 36.

**Fig. 2:**
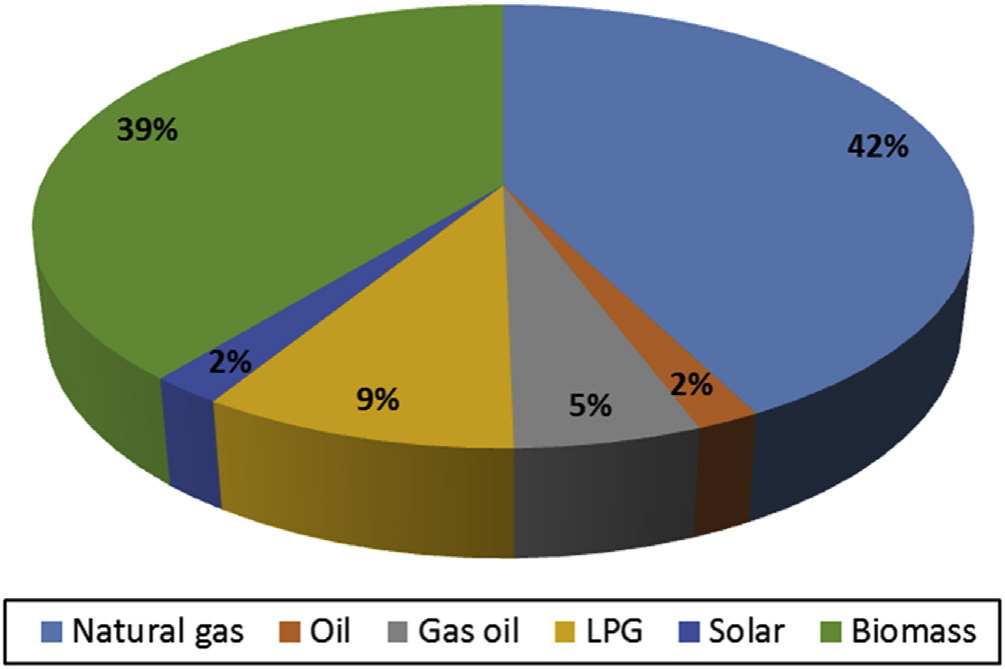
Thermal energy consumption per fuels referred to residential and tertiary sectors in 2013.

Also, the different energy sources used in the mountain community to produce thermal energy for residential and tertiary sectors were analysed in Fig. 2: most of the supply energy comes from natural gas (42%), and biomass (39%); LPG (9%) and the renewable sources, such as the solar one; biofuels are less used. In Piedmont Region natural gas is the principal energy source but in the mountain territories wooden biomass is very used especially for space heating with small boilers (i.e. < 200 kW) in single buildings but the potential expansion of this energy source is very high. In fact, biomass is closely linked to the territory that produces and uses it, thus with more efficient biomass boilers is possible to create a virtuous environmental, economic and social process, favouring also local development.

**Fig. 3:**
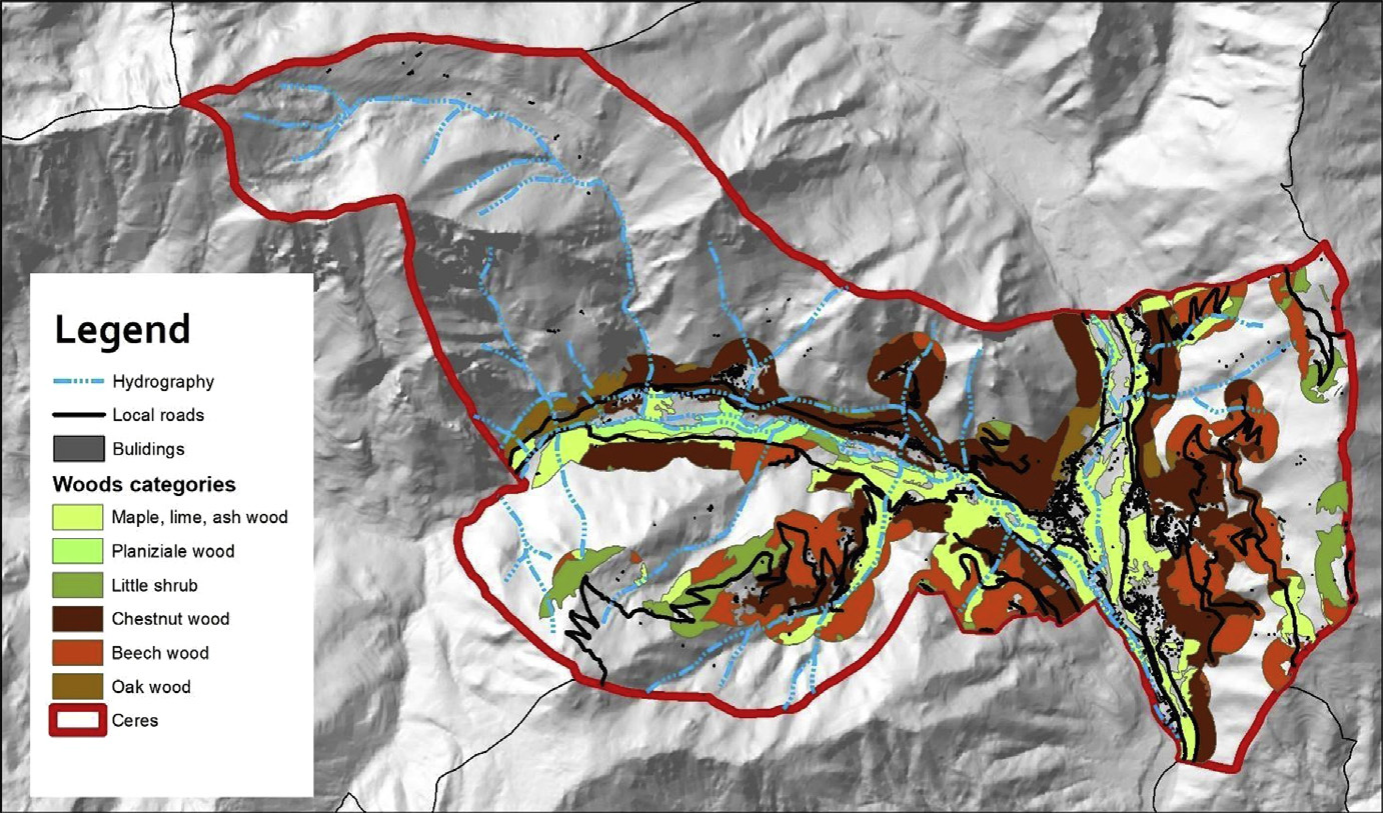
Example of forest biomass resources in Ceres municipality.

Then, in order to reduce the greenhouse gas (GHG) emissions for future low-carbon scenarios, the local availability of woody biomass was also taken into account. In Fig. 3, the different forest biomass resources are represented considering also their accessibility with roads and the territory slope. The analysis conducted in this work starts from previous researches on the evaluation of energy produced by the biomass energy source [14, 15].

In Italy there are 66 unions of mountain municipalities and other 86 unions of hilly municipalities to which this proposed methodology could be applied.

## 4 Materials and methods

**Fig. 4:**
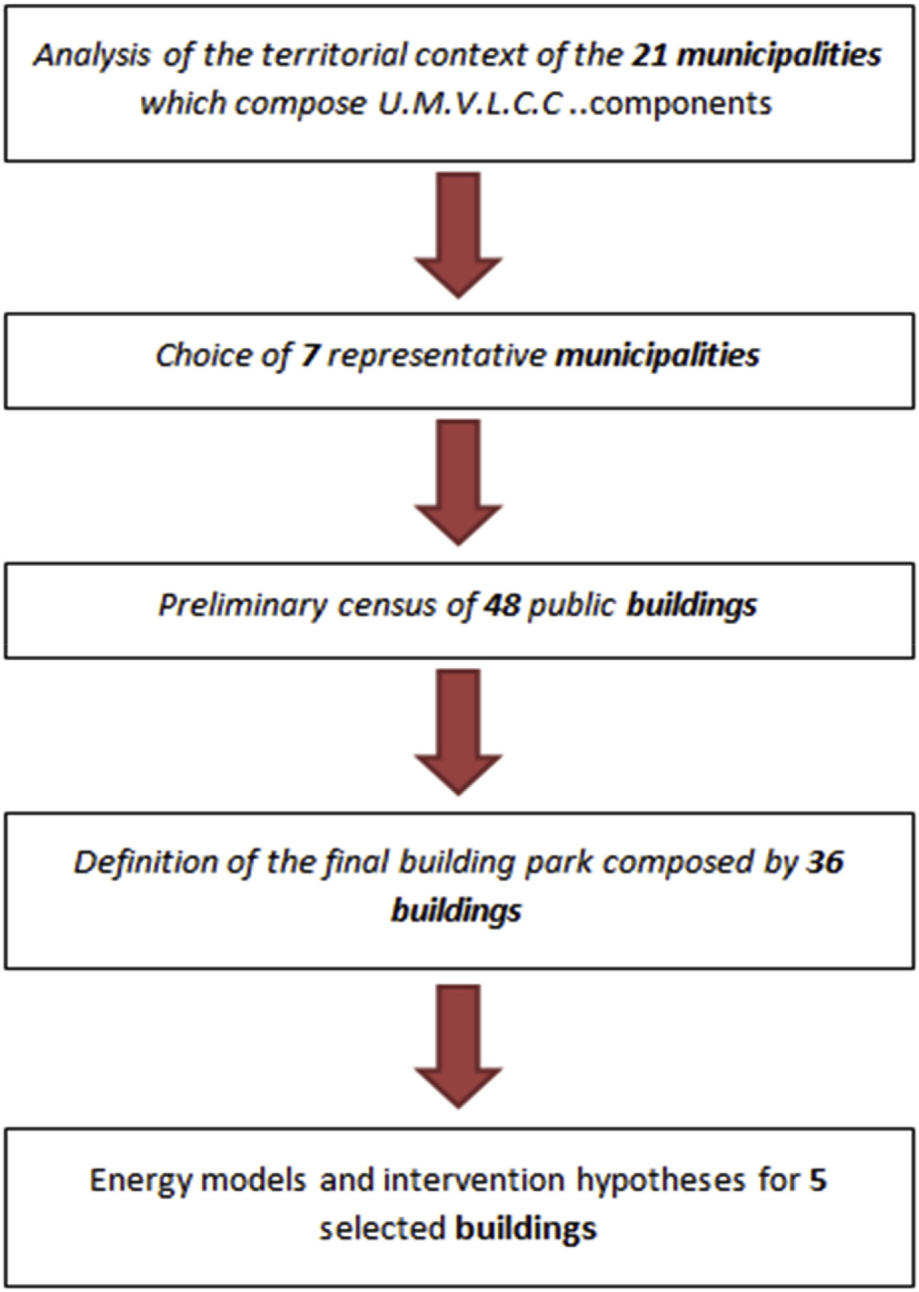
Scheme of adopted methodology in Unione Montana Valli di Lanzo, Ceronda e Casternone (U.M.V.L.C.C.).

**Fig. 5:**
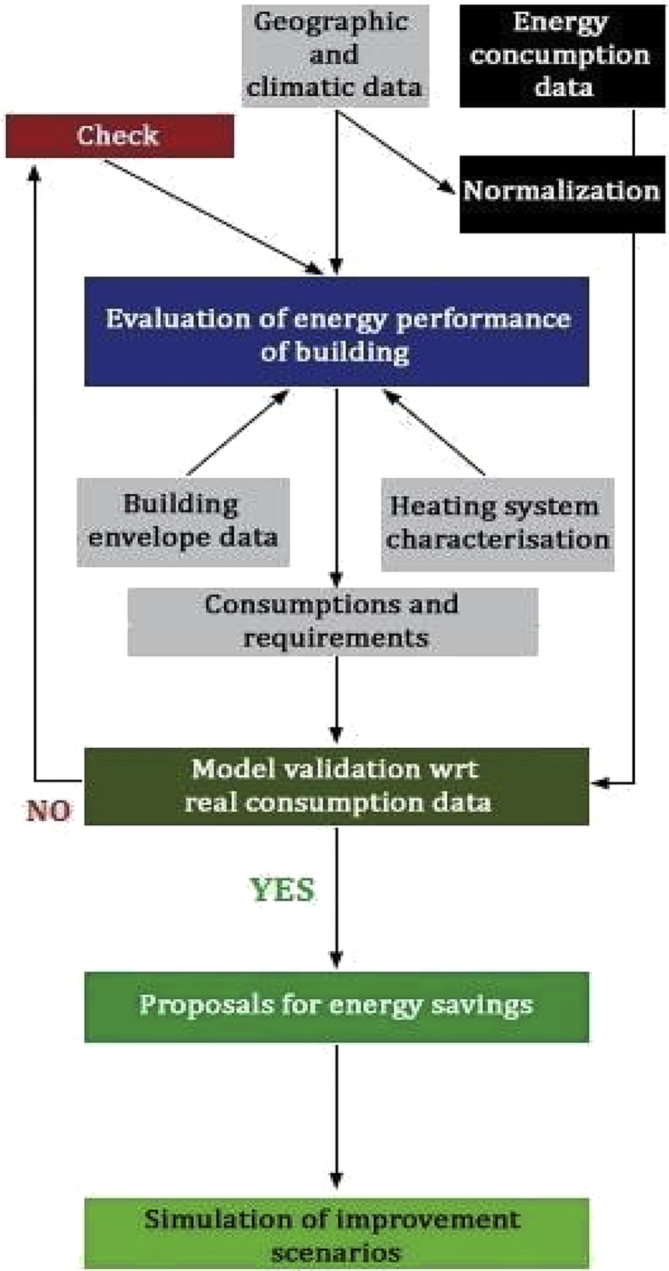
Methodology to identify low carbon and energy efficiency scenarios.

In Figs. 4 and 5, the adopted methodology is presented though the case study of the Mountain Union of Lanzo, Ceronda and Casternone Valleys. As mentioned above, after the analysis of the vast and not homogeneous territory with 21 municipalities and, the selection of 7 representative municipalities, data about 36 public buildings have been analysed.

The information about the sample of public buildings were collected in an energetic cadastre with two types of information: the geometric and typological properties of buildings envelope (with real data or using Italian Standard UNI/TR 11552:2014 [16] knowing the period of construction of buildings), the characteristics of the space heating and hot water production systems, and the data about thermal energy consumptions for the years 2013, 2014 and 2015.

Geometric features of the buildings were used to evaluate surfaces, heated volumes and the surface to volume ratio (S/V) of each building. To obtain these data, existing documents related to renovations or enlargements of public buildings have been analyzed. In the absence of these data, thematic and technical maps with ISTAT census data were examined through the use of a GIS tool. This procedure consents also to compare the energy performance of the different types of buildings to create simplified bottom-up models characterizing the building heritage at territorial scale [7].

In this work, the buildings annual thermal consumptions were normalized through the gross heated volume (kWh/m^3^/y), in order to compare energy efficiency level of public buildings excluding the dimensional component.

The space heating consumptions were calculated considering the three years 2013, 2014 and 2015 with three average variables:

• the annual heating consumption (kWh/y)
• the specific annual consumption (kWh/m^3^/y) and
• the specific annual consumption (kWh_N_/m^3^/y) normalized on the HDD of Lanzo Torinese in order to evaluate the level of energy efficiency of different buildings located in various municipalities regardless of different climatic conditions.

Referring to the consumptions related to the three years 2013, 2014 and 2015, a graphical comparison between annual consumption and the specific annual consumption has been used to identify the most “energivorous” buildings. This graphical representation, called “quadrant method”, allowed the identification of buildings with a higher priority for intervention considering the average value of consumptions for a group of buildings.

Space heating energy consumptions were also normalize with the heating degree days, conventionally at 20 °C, to disregard climatic differences. In Italy, the HDD at 20 °C are also used to define 6 climatic zones (from the warmest A to the coldest F) and, in the analysed mountain community, only La Cassa, Givoletto and Fiano are in the climatic zone E (2101-3000 HDD), while all the other municipalities belong to climatic zone F (with more than 3000 HDD).

To evaluate the critical buildings, in term of energy consumptions and then give a priority of intervention for energy efficiency measures, the annual energy consumption (kWh/y) was compared with the specific energy consumption (kWh/m^3^/y) characterizing respectively the energy costs and the building energy efficiency level. The graphical method used to represent the annual consumption and the specific annual consumption highlights 5 critical buildings with higher energy consumptions.

For each of the 5 critical buildings, a thermal model based on current standard regulations [17, 18, 19] has been implemented with the aim of defining a energy efficiency strategy with its specific financial plan. The results of the energy models were compared with the real energy consumptions according to the methodology shown in Fig. 5 for each building. To define and evaluate the energy performance of the critical buildings, the following parameters have been considered:

• climate with monthly average air temperatures from the reference weather stations for the three years (2013, 2014 and 2015);
• type of building and real occupation time schedules;
• building geometry and type of opaque and transparent envelope with the relative areas and thermal transmittances;
• space heating, hot water production and artificial lighting systems characteristics.

Thermal models were validated by a comparison of the model results with the real energy consumption data, according to Standards on energy audit [18, 19] considering acceptable a relative difference of ±10%. Afterwards, these models were also used to evaluate retrofit interventions in a cost/benefit analysis.

The low-carbon scenarios have been evaluated with the cost-optimal analysis (Italian Standard UNI EN 15459:2008 [20]), defining a comparative framework to identify the optimal cost-based retrofit measures [21]: best scenarios reach low energy consumptions (low EP, kWh/m^2^/y) with low costs (€/m^2^) and these scenarios are localized around the minimum of the curve of cost-optimal graph.

For this evaluation, the following main aspects have been considered:

• Reference prices for retrofit interventions in the Piedmont Region 2016 [22], using also real data from public building retrofit interventions at 2015;
• Financial incentives in Italy for energy efficiency retrofit measures of public buildings (Italian “Conto Termico 2.0:2016”);
• Available annual quantity of wooden biomass, in order to enhance interventions using this local and renewable energy source.

In order to compare the different scenarios, the following parameters for each intervention were calculated:

• Cost of intervention with and without financial incentive (€);
• Energy performance achieved (kWh/m^3^/y);
• Annual energy and economic savings (€/y);
• Payback time of investments (y).

For the cost-optimal analysis, in order to highlight and to exploit scenarios based on the use of renewable sources (particularly biomass), the non-renewable energy performance index EP_gl,nren_ was used to better visualize the energy performance improvements from adoption of renewable energy sources. Then, three cost-optimal analyses have been implemented: the first, considering the total price of the retrofit interventions; the second using current Italian financial incentives and the third excluding the biomass costs for profit and transport, since it was considered a public resource.

The greater use of the local wooden biomass to produce energy in mountain communities in also one of the objective of the Environmental Report of the Regional Air Quality Plan [23]. In fact, in the Italian mountain areas, the use of polluting and inefficient fireplaces especially for space heating of residential buildings, represents a widely widespread reality and their substitution with new efficient biomass boilers could improve air quality with also a reduction of heat dispersions.

Not all the wooden biomass present in the municipal territory is accessible and available; these characteristics depend mainly by: the presence of streets and the slope of the territory influencing the accessibility of a wooden area. To evaluate the quantity of wooden biomass that can be harvest every year to produce energy, a GIS tool with the following databases have been used:

• Piedmont forest map (Sistema Informativo Forestale Regionale, SIFOR) to estimate the tree types and their spatial extension on the territory,
• roadways map and the Digital Terrain Model (DTM) to evaluate the quote of accessible wooden area.

Using a GIS tool it was possible to classify the accessibility of the wooden area around the roads considering the different slope of the terrain. This analysis takes into account the current technologies used in Piedmont Region to collect biomass in territories with different slopes.

At the end of the analysis it was found that in Ceres 900 hectares of wooded biomass can be exploit for the production of energy (Fig. 3), while in Lanzo Torinese only about half. In Table 2 the availability of wooden biomass to produce energy for the two municipalities is reported. This evaluation has been made by a comparison between the accessible wood areas and the database on biomass to produce energy in Regione Piemonte at 2013. Even if Lanzo Torinese is the principal municipality, since it is located in the hillside, has lower biomass availability if compared to Ceres in the mountain area. For the municipalities of Lanzo Torinese and Ceres, the results reported in Table 2 indicate also that the thermal energy produced by biomass could be sufficient to meet the heat energy-use of public buildings.

**Table 2:** Annual availability of wooden biomass (mass of dry wood, kg_dw_) in the Ceres and Lanzo Torinese.

Municipality	Chestnut wood	Maple, lime, ash wook,	Oak wood	Little shrub	Beech wood	Planiziale wood	Robineti wood	Re-forestation	Thermal energy production with biomass	Energy-uses of public buildings
	kg_dw_	kg_dw_	kg_dw_	kg_dw_	kg_dw_	kg_dw_	kg_dw_	kg_dw_	MWh/y	MWh/y
Ceres	1,120,416	508,319	27,598	10,366	237,721	38,406	–	–	7,853	392
Lanzo Torinese	435,403	10,464	73,820	7,974	–	–	102,572	31,825	2,724	1,640

**Fig. 6:**
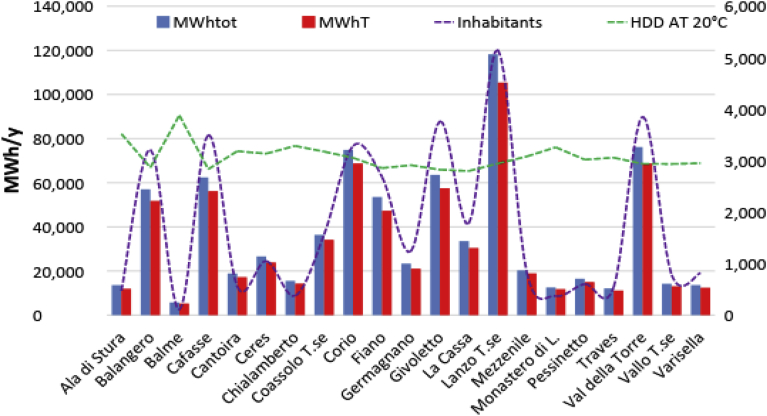
Energy consumption (MWhtot) and thermal energy (MWhT) consumptions for the municipalities of the mountain community of Lanzo, Ceronda and Casternone Valleys with the representation (on the right vertical axis) of the number of inhabitants and the HDD at 20 °C from Standard UNI 10349-3:2016.

As it is possible to observe in Fig. 6 the total energy consumption (thermal and electric components) and thermal energy consumptions depend mainly by the number of inhabitants and the heated volume of buildings besides the climate. The database about the annual per capita thermal energy consumption at the municipal level, provided by the Metropolitan City of Turin for the years 2000–2013 [24], shows that the mountain areas (as Ala di Stura, Cantoira and Ceres) registered higher consumptions if compared with the municipalities located in the plain area. As an example, the annual per capita energy consumption in the municipality of Balme is about 60 MWh/cap/y, more than twice the average of the 21 municipalities belonging to the mountain community analysed (25 MWh/cap/y); as a reference, the City of Turin registered, in the plain area, an energy consumption of about 13 MWh/cap/y. These results are also due to the high presence of holiday houses in the mountain areas and then a lower number of inhabitants.

## 5 Results

In this paragraph the main results of the application of the quadrant graphical method and the cost-optimal analysis on public buildings are presented.

**Fig. 7:**
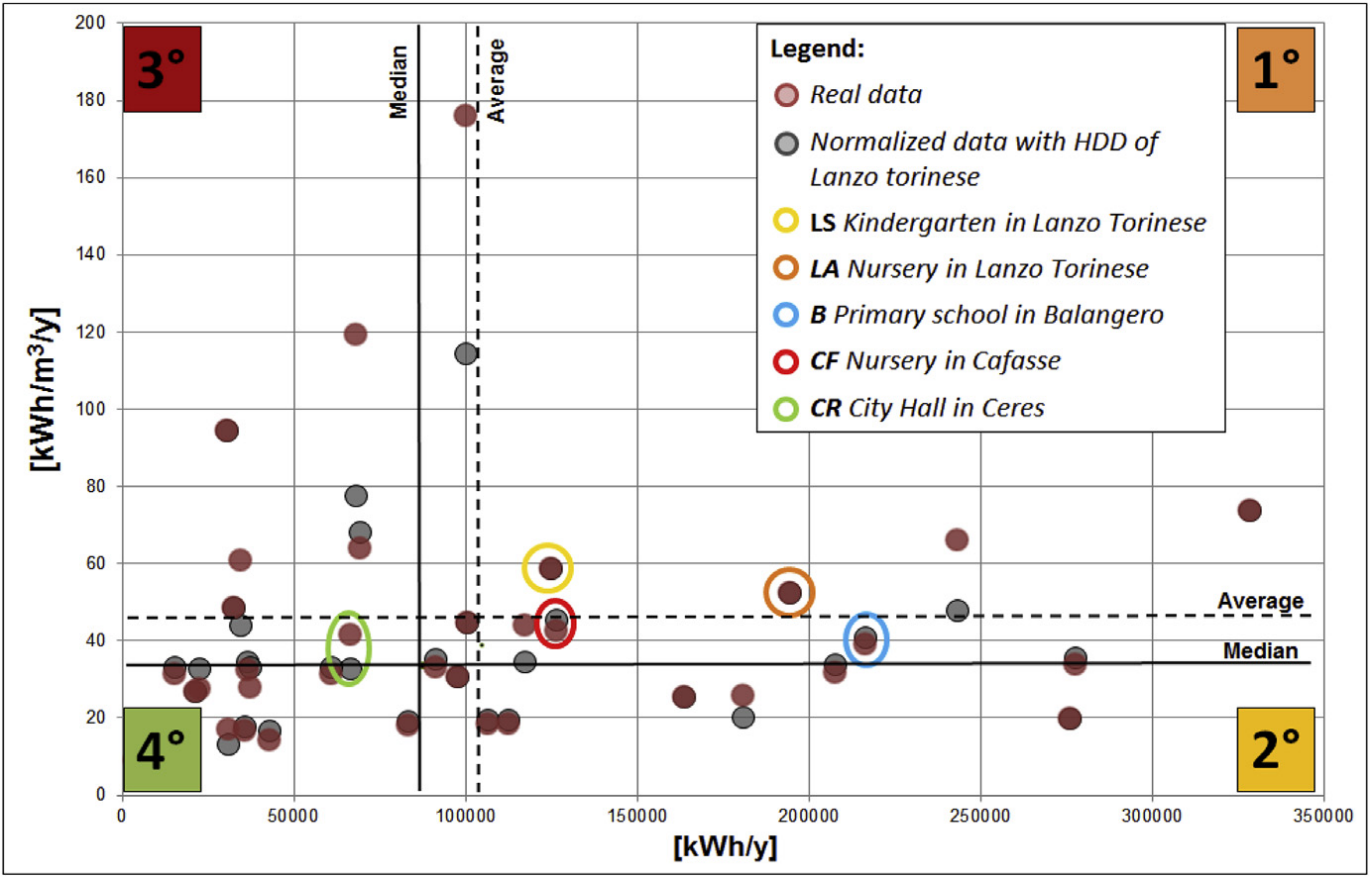
Comparison between annual consumption and the specific annual consumption with the Quadrant Method (B = Primary school in Balangero; LA = Nursery in Lanzo Torinese; LS = Kindergarten in Lanzo Torinese; CF = Nursery in Cafasse; CR = City Hall in Ceres).

In Fig. 7, the quadrant method has been applied to the public buildings of the mountain union; each building is represented by a point, identified by the value of its annual average consumption (x-axis) and its annual average specific consumption (y-axis). A twofold analysis has been implemented:

• in the first case, the traditional graph with the average absolute (kWh/y) and specific (kWh/m^3^/y) energy consumptions of the buildings was represented;
• in the second case, the average absolute consumption (kWh/y) and the specific consumption normalized on 3197 HDD at 20 °C of Lanzo Torinese (kWh_N_/m^3^/y) were represented to evaluate the energy efficiency level as independent by different climatic conditions.

From the quadrant graph illustrated in Fig. 7 it is possible to note the differences between the two analyses and the effects of the HDD normalization on the specific consumption data. With the normalization on HDD, minor values on the specific energy consumption are represented especially for cold climates. The horizontal and the vertical lines represent the average and median values of absolute and specific thermal consumptions of the public buildings sample; in this work, the median value, instead of the average value, was chosen as the reference limit to prevent the presence of anomalous consumption data.

Buildings in the first quadrant have an annual and specific annual consumption higher than the median value, therefore they are buildings with highest energy costs (high kWh/y) and lowest energy performance (high kWh/m^3^/y). The buildings on the fourth quadrant, however, show lowest consumptions both in absolute and in specific terms, than improvements in energy efficiency could not be a priority.

**Fig. 8:**
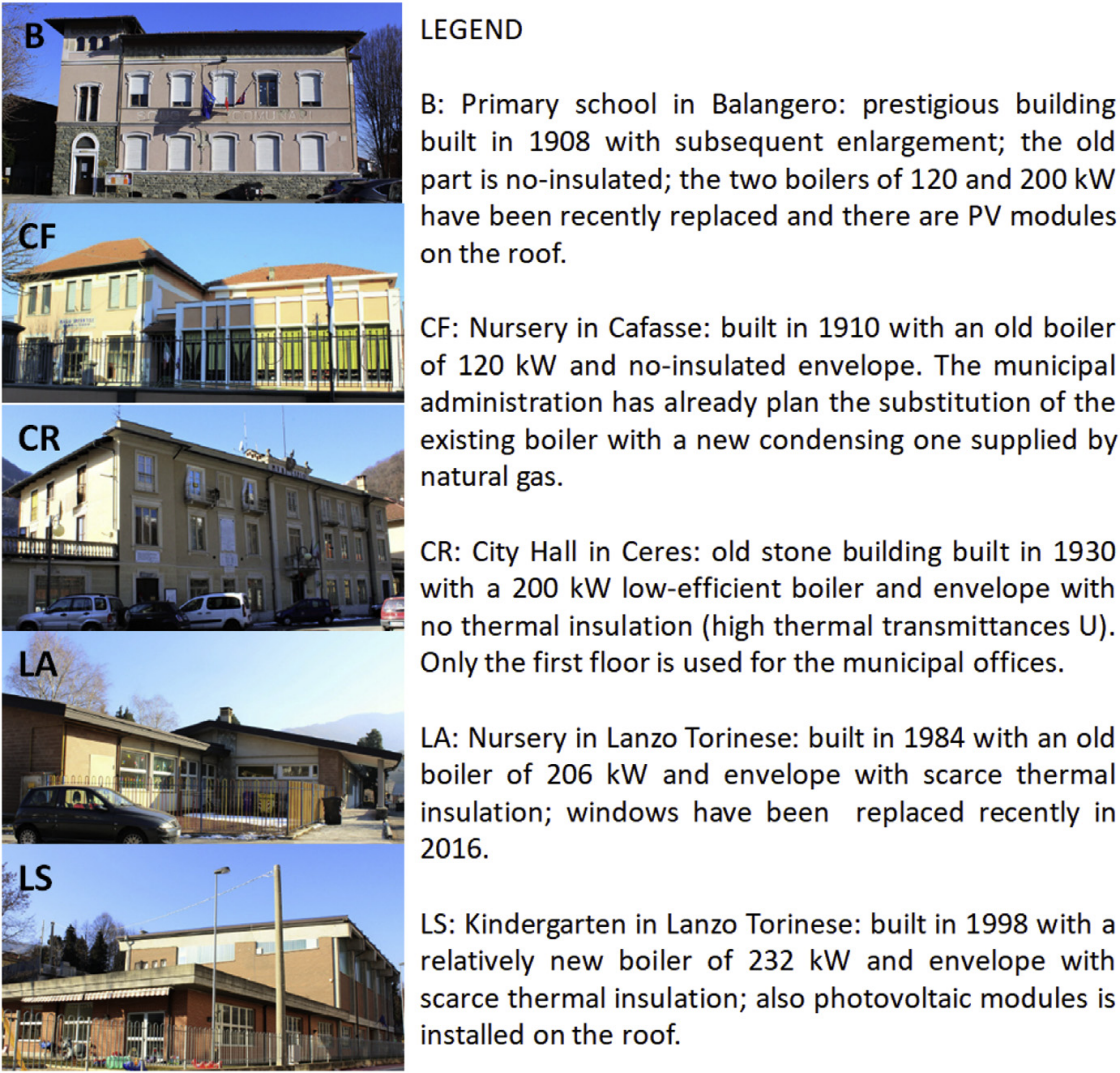
Description of the five critical public buildings.

With this representation, 5 critical buildings have been identified and on these buildings an energy model have been performed to evaluate the effect of energy efficiency measures (Fig. 8). The selected buildings for the cost/benefit analysis of retrofit interventions were: Lanzo Torinese kindergarten, Lanzo Torinese nursery, Balangero primary school, Cafasse nursery and Ceres City Hall. The choice of the buildings has also been agreed with the mountain community taking into account also ordinary maintenance requirements as for Ceres City Hall. For this building, the energy consumption seems low but this value is due to the partial period of operation of the City Hall in a very small municipality like Ceres.

The results of thermal models have been compared to the real energy consumption data for all the buildings and they were considered validated as the relative difference was lower than ±5% (in Table 3). All the buildings were powered by natural gas while, in Ceres, the City Hall has a gas oil boiler.

**Table 3:** Energy-use models results for critical buildings and comparison with real data (considering average energy-use values for the 3 heating seasons).

Building	Calculated consumption	Real consumption	Relative difference
LA: Nursery in Lanzo Torinese	14388 m^3^	13684 m^3^	4.89%
B: Primary school in Balangero	21896 m^3^	22520 m^3^	2.85%
LS: Kindergarten in Lanzo Torinese	20230 m^3^	20122 m^3^	0.53%
CF: Nursery in Cafasse	12713 m^3^	13181 m^3^	3.68%
CR: City Hall in Ceres	6536 l	6559 l	0.35%

The proposed efficiency retrofit measures have considered interventions on building envelope and heating system considering the existing Italian Law requirements (Italian Decree D.M. 26/6/2015); then, the following interventions have been adopted:

• thermal insulation of building opaque envelope (walls, slabs and roofs),
• replacement of windows,
• replacement of boiler with a condensing one using natural gas or biomass,
• installation of thermostatic valves and
• installation of solar collectors and/or photovoltaic systems.

A total of 58 retrofit scenarios have been implemented, some with individual interventions and others aggregating individual ones, as it is illustrated in Table 4. Retrofit interventions are different for each buildings because not all interventions are technically and economically feasible; as an example, for the school in Balangero, no biomass and PV systems have been provided since there were new boilers with relatively high efficiencies.

**Table 4:** Retrofit interventions *For to the part of building built in 1908; ** For the part of building built in 2000.

Building	Interventions
Primary school in Balangero	Windows replacement *(B1), Windows**(B2), External walls insulation*(B3), External walls insulation**(B4), B1 + B3, B3 + B4, B1 + B2, B1 + B4, B1 + B2 + B3 + B4
Nursery in Lanzo Torinese	External walls insulation (LA1), Roof insulation (LA2), Floor insulation (LA3), LA1 + LA2, Generator replacement (natural gas) (LA5), LA5 + LA4, Installation biomass generator (LA7), LA7 + LA1, LA7 + LA2, LA7 + LA1 + LA2
Kindergarten in Lanzo Torinese	Roof insulation (LS1), Windows replacement (LS2), External walls insulation (LS3), Generator replacement (natural gas) (LS4), LS1 + LS3, LS1 + LS4, LS1 + LS2 + LS3, LS1 + LS2 + LS3 + LS4, Installation biomass generator (LS9), LS1 + LS2 + LS3 + LS9, LS1 + LS9, LS3 + LS9, LS2 + LS9
Nursery in Cafasse	Slab insulation (CF1), Windows replacement (CF2), External walls insulation (CF3), Photovoltaic technology installation (CF4), Solar technology installation (CF5), CF1 + CF2, CF1 + CF3, CF1 + CF2 + CF3, CF1 + CF3 + CF5, CF2 + CF3 + CF5, CF1 + CF2 + CF3 + CF4 + CF5
City Hall in Ceres	Floor insulation (CR1), External walls insulation (CR2), Windows replacement (CR3), CR1 + CR2, CR2 + CR3, CR1 + CR2 + CR3, Installation biomass generator (CR7), CR7 + CR1 + CR2, CR2 + CR3 + CR7, CR1 + CR2 + CR3 + CR7

In order to define the global cost of retrofit interventions, some parameters have been calculated:

• the life period of retrofit measures,
• the discount rate and
• the investment costs: for the design, purchase, installation, annual maintenance and energy costs.

In this study, the global costs were composed by the sum of all costs projected over a 30 years of life period using a 0.3% of discount rate; the cost was divided by the net heated area of each building. The cost of annual energy for space heating was calculated as a product between the annual thermal energy-use of the building and the cost of the fuel used (in Table 5), while the cost of the interventions was calculated with and without the current financial incentives for building energy retrofit.

**Table 5:** Conversion coefficient for primary energy and fuel costs in Italy.

Fuel	Conversion coefficient for primary energy	Fuel cost [€/kWh]
Natural gas	1.05|_nren_	0.082
Gas oil	1.07|_nren_	0.102
LPG	1.05|_nren_	–
Electric energy	1.95|_nren_ and 0.47|_ren_	0.200
Biomass	0.2|_nren_ and 0.8|_ren_	0.27^a^|0.081^b^
Solar	1.0|_ren_	–

a included in the cost: biomass, firewood and woodchips purchase and transport.

b cost of biomass considered as a public and local resource managed by the mountain community: low costs associated to profit and transport.

In this work, the specific energy performance achieved was defined with the global primary energy performance index EP (kWh/m^2^/y) given by renewable and not renewable energy sources:

(1) EP_gl_ = EP_gl,ren_ + EP_gl,nren_.

To obtain the primary energy, energy consumptions were multiplied by the coefficients of conversion into primary energy, specified in the Italian Decree D.M. 26/6/2015. In Table 5, it is possible to observe that 20% of solid biomass is not renewable.

**Fig. 9:**
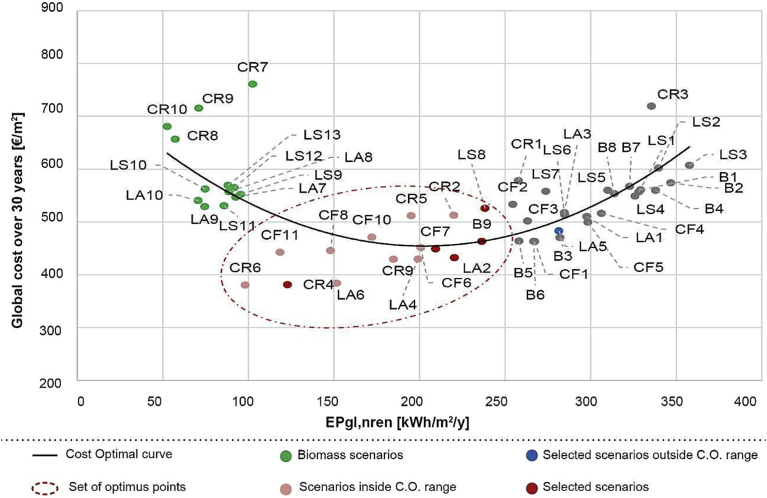
Cost optimal analysis without financial incentives (B = Primary school in Balangero; LA = Nursery in Lanzo Torinese; LS = Kindergarten in Lanzo Torinese; CF = Nursery in Cafasse; CR = City Hall in Ceres).

**Fig. 10:**
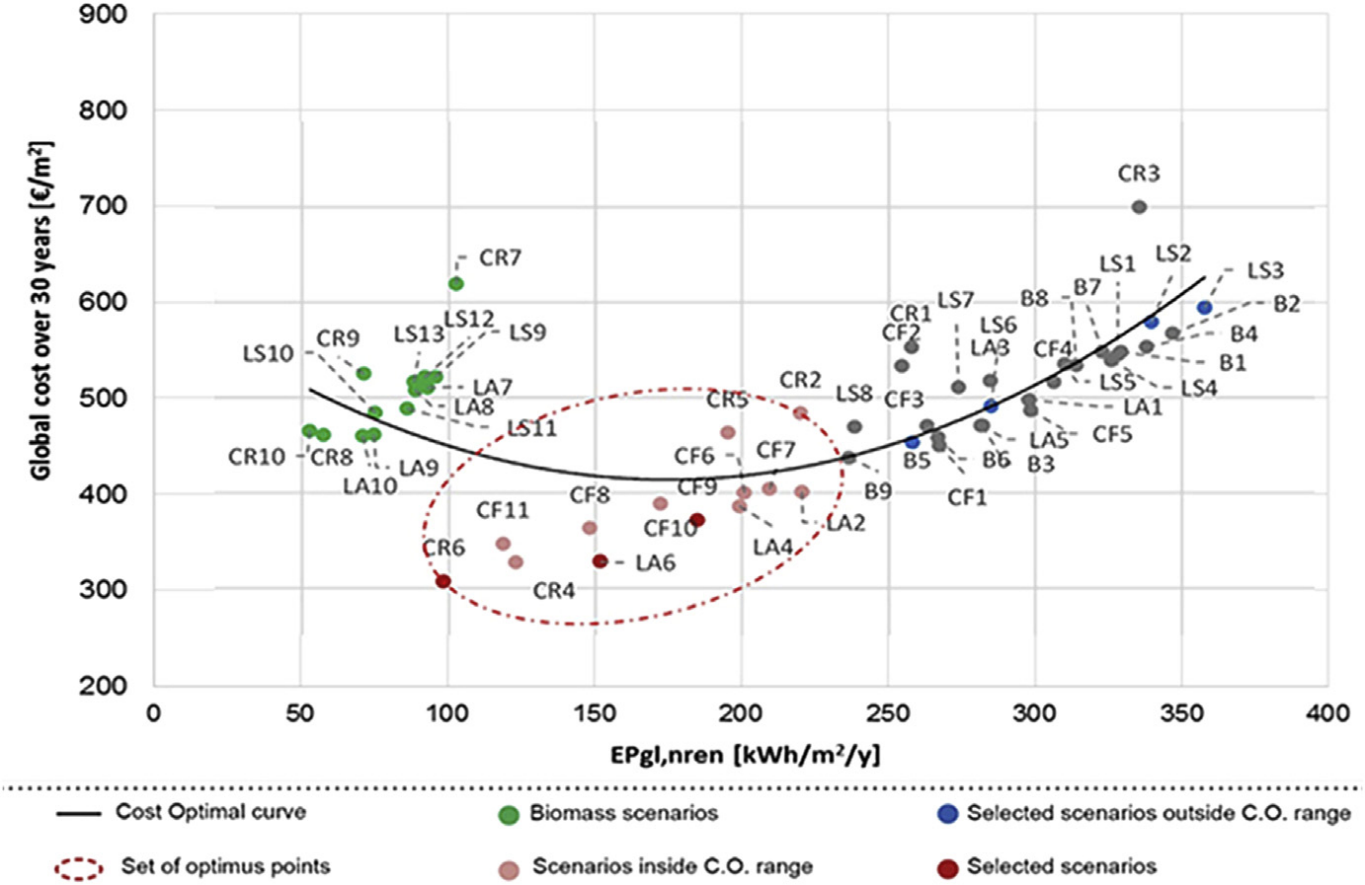
Cost optimal analysis applying financial incentives (B = Primary school in Balangero; LA = Nursery in Lanzo Torinese; LS = Kindergarten in Lanzo Torinese; CF = Nursery in Cafasse; CR = City Hall in Ceres).

**Fig. 11:**
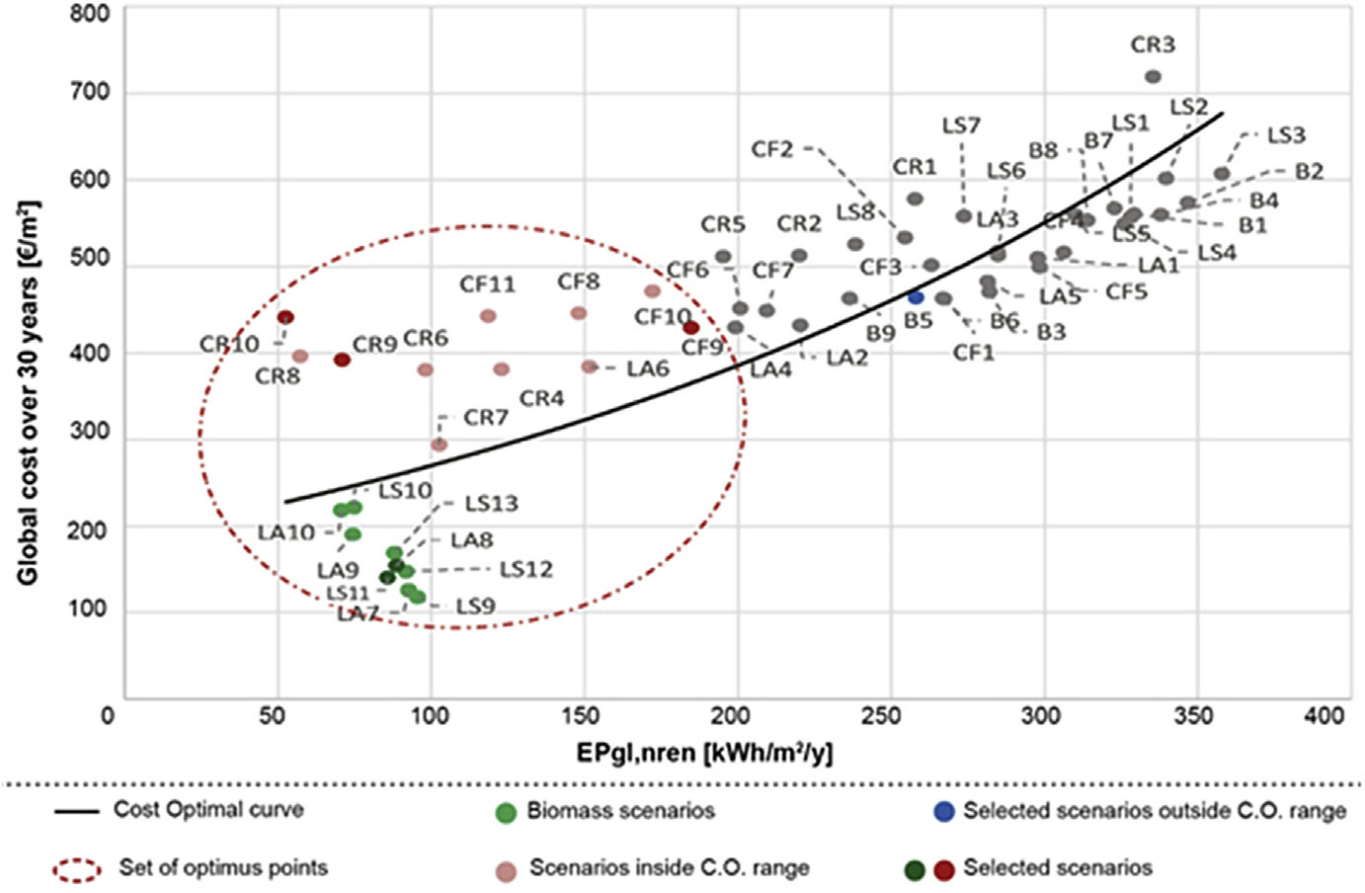
Cost optimal analysis with financial incentives and local available biomass (B = Primary school in Balangero; LA = Nursery in Lanzo Torinese; LS = Kindergarten in Lanzo Torinese; CF = Nursery in Cafasse; CR = City Hall in Ceres).

In Figs. 9, 10, and 11, the three different cost-optimal analyses are represented. These graphical representations are useful for highlighting interventions characterized by a good compromise between global costs and final primary energy performances. These better interventions are located in the minimum of the curves with low global costs and low primary energy consumption EP. Each intervention is identified by an alphanumeric code were the letters represent the building under analysis and the numbers the type of retrofit measure (as in Table 4).

In the cost-optimal analysis, represented in Figs. 9 and 10, it is possible to notice how the scenarios based on biomass are characterized by optimal low values of EP, but higher costs (with a cost of biomass of 0.27 €/kWh). In fact, these scenarios take into account the higher cost associated to a biomass boiler on the market, with respect to a traditional one. The optimum scenarios, highlighted in these two analyses, are mostly based on the insulation of opaque envelope accomplished by the substitution of obsolete boiler (i.e. CR6, LA6, B9 and CF5).

The third cost-optimal analysis, represented in Fig. 11, excluded the costs associated to profit and transport of biomass, since this resource is directly managed by the mountain community. In this case, the global cost of biomass was reduced by the 30% and the optimal cost curve drastically changes, highlighting the scenarios based on biomass fuel.

For each of the scenarios proposed by the cost-optimal analysis, a financial plan was defined taking into account the specific interventions, in order to reinvest the economic savings obtained from previous energy saving interventions for each building.

**Fig. 12:**
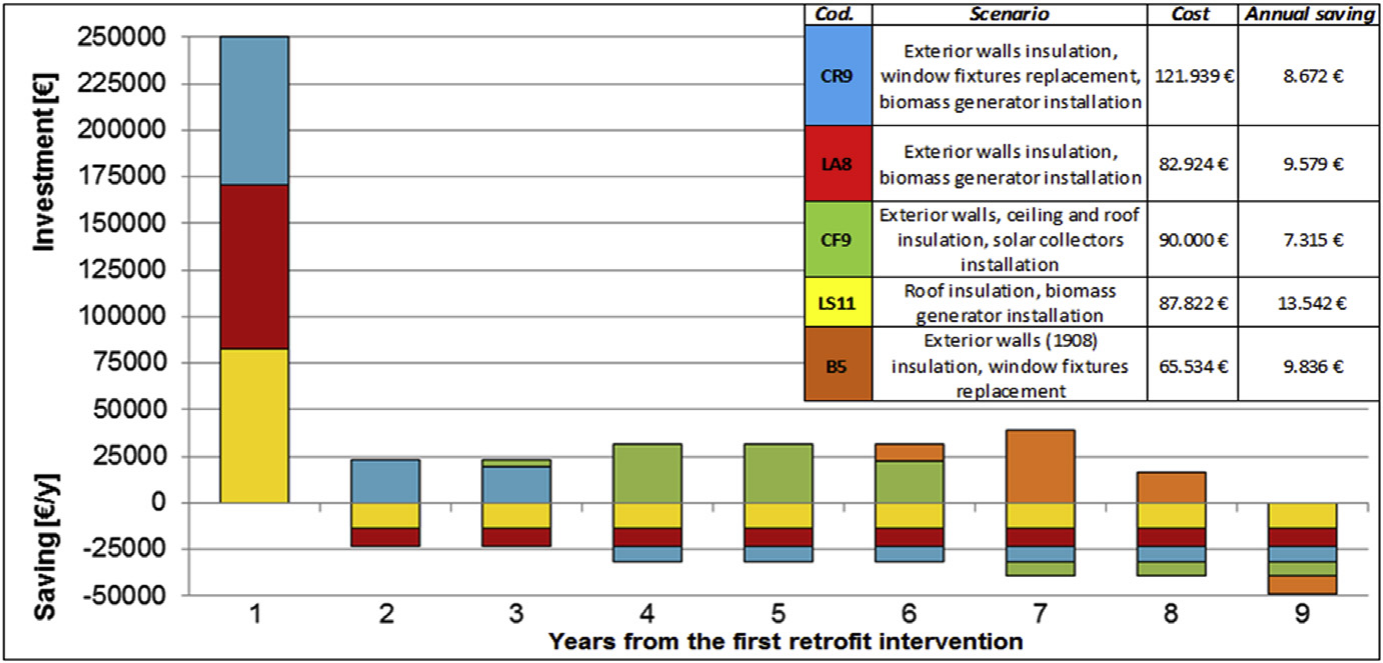
Financial plan of selected retrofit interventions, referred to cost-optimal analysis with financial incentives and local available biomass.

In Fig. 12, the financial plan referred to the better retrofit scenarios is represented considering financial incentives and local availability of biomass. In this case, a starting budget of 250,000 € was considered to allow the adoption of the more convenient scenario (derived by cost-optimal analysis). The focus is to invest the economic savings implemented in the previous year in further retrofit interventions for all the select buildings. After 9 years, it is possible to invest 50,000 € in other buildings retrofit; it should be considered also that this economic saving will be forever.

## 6 Discussion

This work provides an accurate methodology that can be replicated in other territorial contexts to evaluate the effects of different policies for a more sustainable development. The methodology adopted, in fact, has allowed to effectively utilize existing methods, adapting them to a specific case study as the Lanzo, Ceronda and Casternone Valleys Union.

The presented approach has taken into account the complexity and specificity of this mountain territory, considering the more effective energy efficiency interventions, with an analysis on energy consumptions on a sample of representative critical buildings. The results can also be extendable to the other public buildings in a similar area. The applied methodologies, in particular the quadrants method and the cost-optimal analysis, have been modified in order to obtain more effective results on the analysed mountain community.

In the quadrant method the annual energy consumption was compared with the specific energy consumption normalized on the HDD. With this approach, even for a vast territory, the level of energy efficiency, measured with the specific energy consumption, does not vary with the climate and depends only by the buildings characteristics. This normalized value of specific consumption can help identifying an order of priority of retrofit interventions to improve energy efficiency, while the annual consumption is not normalized because it represents the annual energy costs.

**Fig. 13:**
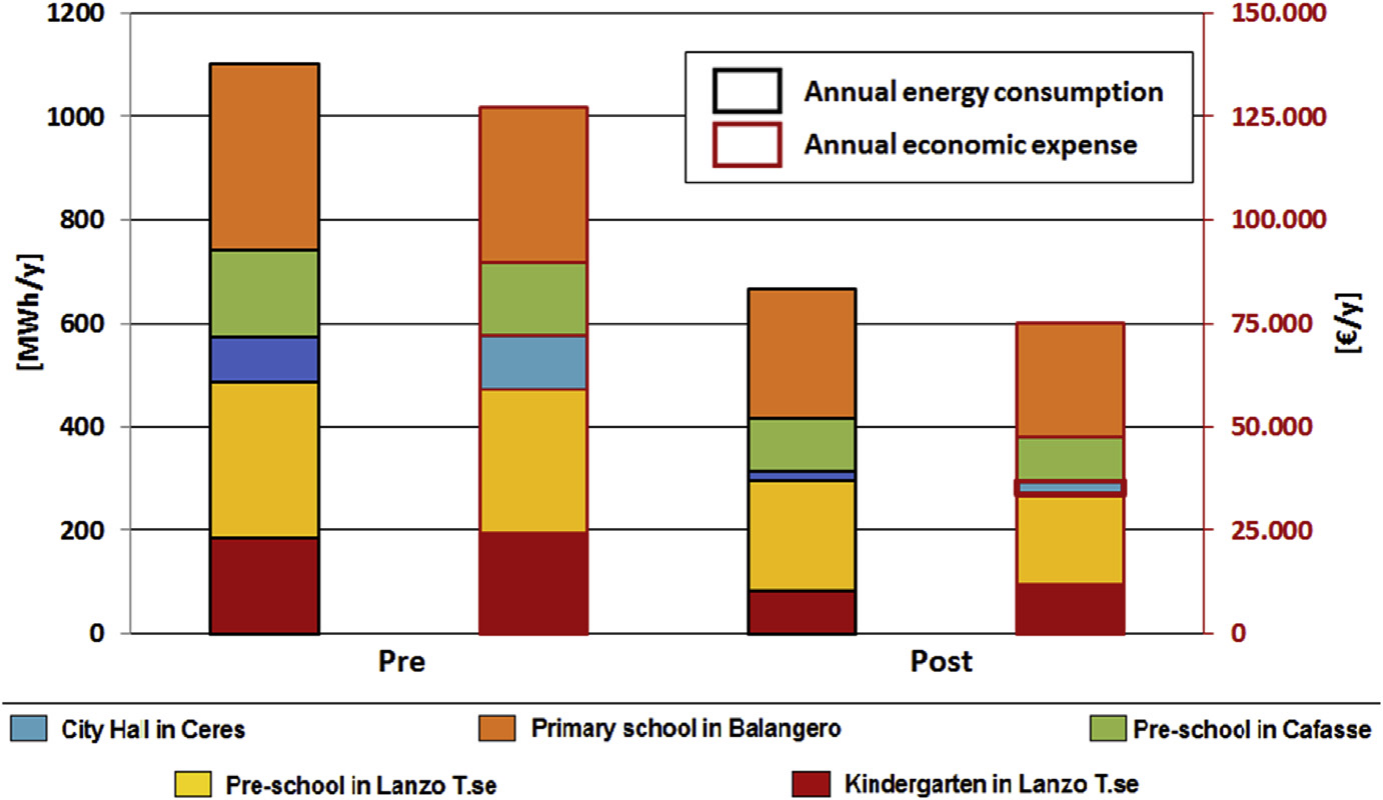
Comparison between annual energy consumption and economic costs pre and post retrofit interventions.

Fig. 13 illustrates the difference between the current energy consumptions of the analysed buildings and the results obtained by implementing retrofit interventions defined in the financial plan with the exploitation of the available biomass resource. The reduction in the energy-use is of about 40% with also a reductions of energy costs of 41%.

**Fig. 14:**
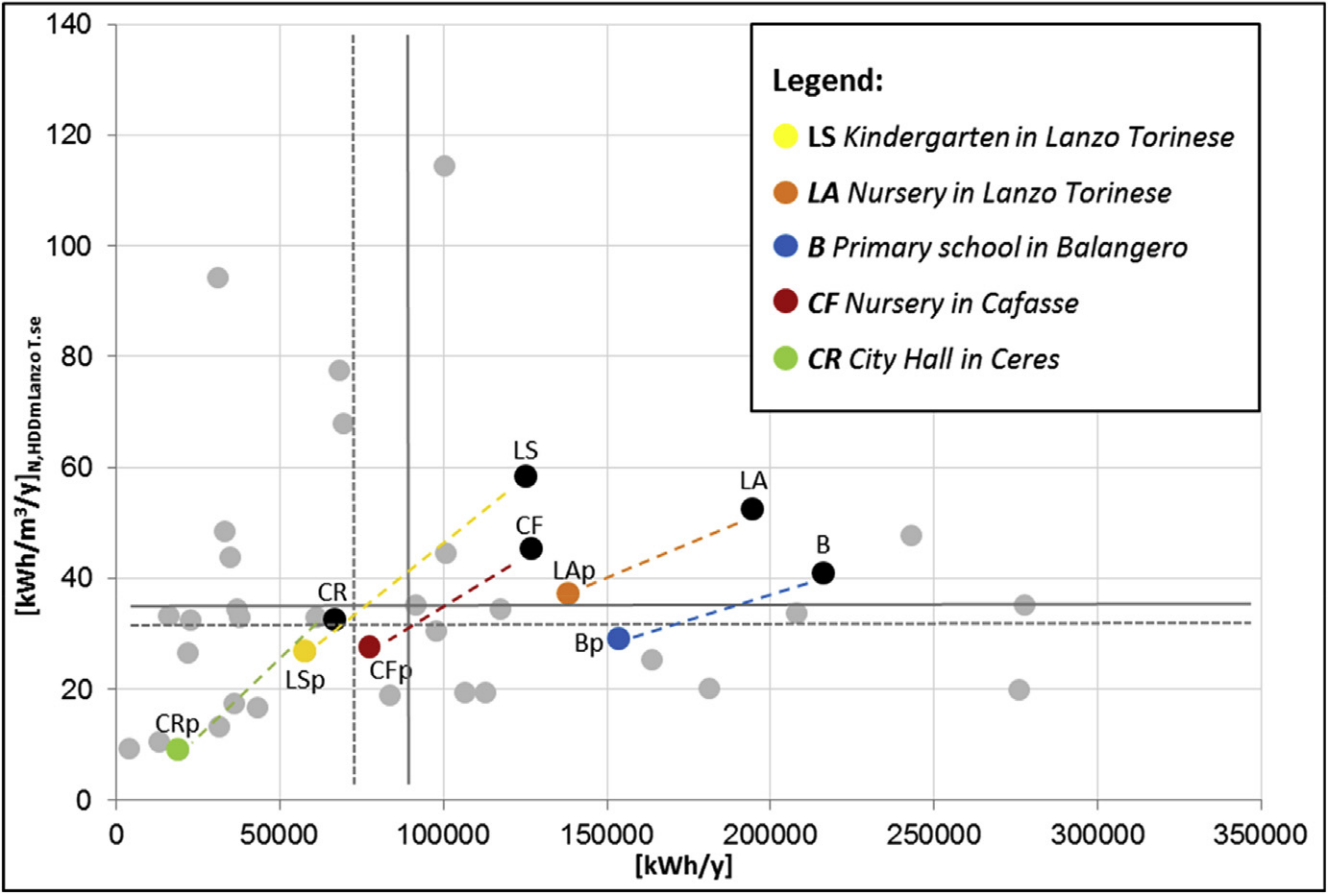
Comparison between annual consumption and the specific annual consumption (Quadrant method) pre and post retrofit interventions.

Fig. 14 is a representation of quadrant method pre and post-retrofit interventions. It is possible to observe a migration of the points representing the energy performance of the analysed buildings in the low-left side of the graph achieving lower values of absolute and specific energy consumption (especially Ceres City Hall, Lanzo kindergarten and Cafasse nursery). The goodness of the retrofit interventions can be measured with the slope of the dotted line that ensures a reduction in annual consumption but also a decrease in specific consumption that measures the level of energy efficiency reached. Balangero primary school moves from the first to the second quadrant, showing absolute consumption above the median value, but a good level of efficiency achieved. Lanzo Torinese nursery offers a general improvement in performance without achieving the optimal quadrant.

From the graphs represented in Figs. 9, 10, and 11, it can be noticed that, over the life period of thirty years, applying a market price to biomass, the scenarios using biomass boilers are uncompetitive and unfavourable; while, taking into account the local availability of biomass, these interventions are also economically convenient. This analysis tries to highlight the retrofit scenarios that include the installation of a biomass boiler in order to exploit a local and renewable resource as an alternative to traditional fuels. For the economic feasibility, this was possible by considering a lower price of biomass as its local availability and the municipal property of the forest areas; thus, the biomass price was reduced by 30% to deduct the costs of biomass profit and transport. Indeed, Fig. 10 shows that currently, the existing economic incentives are not adequate to support the purchase of single biomass boilers but the acquisition of numerous systems for all the community could be investigated, as the installation of cogeneration biomass plants to supply more buildings.

The cost-optimal analysis evidenced that retrofit interventions only on thermal insulation of buildings envelope are not economically convenient, while good performances can be observed if they are combined with the substitution of the boiler. Furthermore, retrofit interventions in the mountain areas are more convenient than in the hilly ones because the investment costs are constant, while the economic savings after retrofits are higher.

## 7 Conclusions

This methodology is answering to the European Cohesion Policy 2014–2020, providing effective actions based and the real building heritage to support the transition to a low-carbon economy and the Urban Agenda Habitat III [25]. Energy sustainability and security is one of the milestones of the new Urban agenda and, with regard to Italy, the objectives are clear: reducing energy and primary energy consumptions in buildings and implementing tools to support the transition to a low-carbon economy by promoting a gradual renovation of buildings and the adoption of renewable energy technologies.

The diffusion of efficient biomass systems using the availability of this local resource could lead to a twofold advantage: on the one hand, it consent to contain economic costs for public administrations, while on the other hand, a virtuous circle of good forest management would be created with a probable economic and environmental benefits for the local communities. Especially for high-income countries like Italy, it is important not only the development and application of low-carbon production technologies but also the promotion of the progress in low-carbon production technologies to reduce the probable scale effect on emissions due to economic growth [26].

The novelty of the methodology is on the application of a GIS-based approach to reach the energy security in a vast and various territory guarantying energy supply where there is energy demand with the available renewable energy sources. This methodology was also supported by effective tools as the quadrant method and the cost-optimal graph to find the more efficient actions. In this work, these tools have been modified to operate at territory scale with different climate characteristics and various building heritages as a mountain community. In particular the energy efficiency level of buildings was normalized on HDD and the energy performance was calculated with the not renewable component of EP, considering the costs with financial incentives and further discounts for the local resources.

Finally, the Piedmont Region is the first region in Italy studying a law to facilitate the creation of energy communities that can self-generate the energy they need by exploiting technologies that produce electricity and heat from renewable sources available locally. This methodology will be tested on the first case studies in future researches.

## Declarations

### Author contribution statement

Guglielmina Mutani, Mauro Cornaglia, Massimo Berto: Conceived and designed the analysis; Analyzed and interpreted the data; Contributed analysis tools or data; Wrote the paper.

### Funding statement

This research did not receive any specific grant from funding agencies in the public, commercial, or not-for-profit sectors.

### Competing interest statement

The authors declare no conflict of interest.

### Additional information

No additional information is available for this paper.
